# Mono- and Binuclear Complexes in a Centrifuge-Less Cloud-Point Extraction System for the Spectrophotometric Determination of Zinc(II)

**DOI:** 10.3390/molecules29184511

**Published:** 2024-09-23

**Authors:** Kiril B. Gavazov, Petya V. Racheva, Antoaneta D. Saravanska, Fatma Genc, Vassil B. Delchev

**Affiliations:** 1 Department of Chemical Sciences, Faculty of Pharmacy, Medical University of Plovdiv, 120 Buxton Bros Str., 4004 Plovdiv, Bulgaria; petya.racheva@mu-plovdiv.bg (P.V.R.); antoaneta.saravanska@mu-plovdiv.bg (A.D.S.); 2 Faculty of Pharmacy, Istanbul Yeni Yüzyıl Üniversitesi, 26 Yılanlı Ayazma Caddesi, 34010 Istanbul, Turkey; ftmgenc@yahoo.com; 3 Faculty of Chemistry, University of Plovdiv ‘Paisii Hilendarskii’, 24 Tsar Assen St., 4000 Plovdiv, Bulgaria; vdelchev@uni-plovdiv.net

**Keywords:** zinc(II), centrifuge-less cloud-point extraction, 6-Hexyl-4-(2-thiazolylazo)resorcinol, spectrophotometric determination, TD DFT, binuclear complex

## Abstract

The hydrophobic reagent 6-hexyl-4-(2-thiazolylazo)resorcinol (HTAR) was investigated as part of a cloud-point extraction (CPE) system for the spectrophotometric determination of Zn(II). In the system, complexes with different stoichiometries, including 1:1 and 2:2 (Zn:HTAR), are formed. Their ground-state equilibrium geometries were optimized at the B3LYP/6-31G level of theory. The obtained structures were then used to calculate vertical excitation energies in order to generate theoretical UV/Vis absorption spectra. The comparison between theoretical and experimental spectra demonstrated that, under optimal conditions, a binuclear complex containing oxygen-bridging atoms is the dominant species. The absorbance was found to be linearly dependent on the concentration of Zn(II) within the range of 15.7 to 209 ng mL^−1^ (*R*^2^ = 0.9996). The fraction extracted (%*E*), logarithm of the conditional extraction constant (log *K*_ex_), and molar absorption coefficient (*ε*) at *λ*_max_ = 553 nm were calculated to be 98.3%, 15.9, and 4.47 × 10^5^ L mol^−1^ cm^−1^, respectively. The method developed is characterized by simplicity, convenience, profitability, sensitivity, and ecological friendliness. It has been successfully applied to the analysis of pharmaceutical and industrial samples.

## 1. Introduction

Zinc is the lightest element in Group 12 of the periodic table. Despite its inclusion in the d-block, it has fully filled 3d orbitals and does not meet the IUPAC definition of a transition metal [1]. These occupied 3d orbitals are energetically stable and remain unchanged during chemical processes. Therefore, unlike typical transition metals, zinc is only able to lose its two 4s electrons, resulting in a stable Zn^2+^ ion.

Zinc is the 24th-most-abundant element in Earth’s crust, with an average content of 65 mg kg^−1^ [2]. It is part of 320 different minerals, representing 5.3% of all minerals in the International Mineralogical Association’s database [3]. The most common of these is sphalerite (ZnS), which is normally found in bimetallic lead–zinc deposits [4]. Elemental zinc is produced primarily through two distinct technologies, namely hydrometallurgy and pyrometallurgy. It is the fourth-most-utilized metal, following iron, aluminum, and copper [2].

It is well established that zinc plays a pivotal role in biological processes and is an essential element for humans, animals, plants, and microorganisms. Zinc deficiency represents a significant public health concern [5], with an estimated 4.4% of childhood deaths in developing countries potentially preventable through zinc nutrition [6]. The recommended daily intake of zinc for adults is 15 mg [7,8]. In contrast to its counterparts in Group 12 (Cd and Hg), it is not regarded as a toxic element [9]. Nevertheless, when consumed in substantial quantities (e.g., 50 mg day^−1^ and above) over an extended period, zinc can cause harm. Toxic effects have been documented in persons exposed to pesticides and compounds used in the manufacture of paints, rubber and dyes. Other instances of intoxication have been attributed to the excessive use of Zn-containing dietary supplements or denture adhesive creams [9,10,11].

A variety of spectroscopic techniques have been employed for the determination of zinc, including atomic absorption spectrometry (AAS), inductively coupled plasma–optical emission spectrometry (ICP-OES), spectrofluorimetry, and spectrophotometry. They can be integrated with a number of pre-concentration techniques in order to enhance analytical performance and expand the scope of application.

Cloud-point extraction (CPE) is a straightforward, cost-effective, and environmentally friendly pre-concentration technique that was developed in 1976 [12] as an alternative to the classical liquid–liquid extraction (LLE). It is based on the partition of the analyte between two phases formed in a micellar system and is regarded as a “green analytical technique” due to the use of non-toxic surfactants in place of large amounts of volatile, flammable, and toxic organic solvents [13,14,15,16].

It is noteworthy that the discoverer of CPE, Hiroto Watanabe, made his breakthrough shortly after conducting a spectrophotometric study of the complexation between Zn(II) and 1-(2-pyridylazo)-2-naphthol (PAN). In his initial publication [17], Watanabe employed a non-ionic surfactant (Triton X-100) as a solubilizer. Subsequently, when the novel CPE technique gained attention due to its advantages, he returned to the Zn(II)–PAN system, proposing the first CPE–UV/Vis approach for the determination of Zn(II) [18]. 

In the following years, a number of CPE methodologies have been proposed for Zn(II) determination. These methodologies employ AAS [19,20,21,22,23,24,25,26,27,28,29], ICP-OES [20,30,31], spectrofluorimetry [32], and spectrophotometry [33,34,35,36,37,38]. However, most of them have inherent limitations, including complexity of implementation, insufficient selectivity, expensive equipment, narrow ranges of optimized parameters, and the necessity for the synthesis of reagents. Moreover, studies of the complexes formed, if conducted at all, have been limited mainly to molar ratio determination.

This paper has three main objectives: (i) to investigate the complexation between Zn(II) and 6-hexyl-4-(2-thiazolylazo)-resorcinol (HTAR) in a CPE system containing Triton X-114 (TX-114); (ii) to determine its suitability for the CPE–spectrophotometric determination of Zn(II); and (iii) to model the complexes formed using theoretical TD DFT calculations. A validating criterion for the conclusions drawn about the composition and structure of the extracted species would be a satisfactory match between the experimentally recorded and the theoretically generated spectra.

HTAR is a commercially available azo dye [39,40] that has recently been applied for the CPE–spectrophotometric determination of vanadium [40], copper [41], cobalt [42], cadmium [43], and mercury [43]. Its complexes are separated easily by gravity, eliminating the need for a centrifuge. This approach, designated “centrifuge-less CPE” (CL-CPE) [13,14,41,42], is slightly slower than traditional CPE but offers a simpler and more energy-efficient alternative.

## 2. Results and Discussion

### 2.1. Absorption Spectra and CPE Optimization

**Figure 1:**
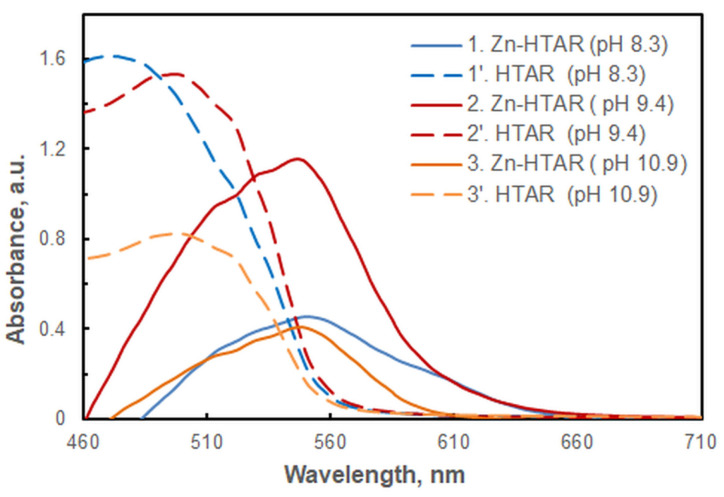
Absorption spectra of the complexes against blank (1–3) and spectra of the corresponding blanks (1–3′) at different pH values: 8.3 (1, 1′), 9.4 (2, 2′), and 10.9 (3, 3′). *c*_Zn(II)_ = *c*_HTAR_ = 6 × 10^−6^ mol L^−1^, *w*_TX-114_ = 1.2%, *V*_buffer_ = 1.8 mL, *t* = 30 min at 50 °C, *m*_SRP_ = 3 g.

The complexation between Zn(II) and azo dyes, such as PAN [18,21,26,33,36], 2-(5-bromo-2-pyridylazo)-5-diethylaminophenol (5-Br-PADAP) [27], Sudan III [37], and 4-(2-pyridylazo)-resorcinol (PAR) [44,45], is known to occur in alkaline media. Similarly, the interaction of Zn(II) with HTAR also requires an alkaline environment. However, our observations indicate that complexes with different spectral properties are formed in response to small changes in experimental conditions (Figure 1).

**Figure 2:**
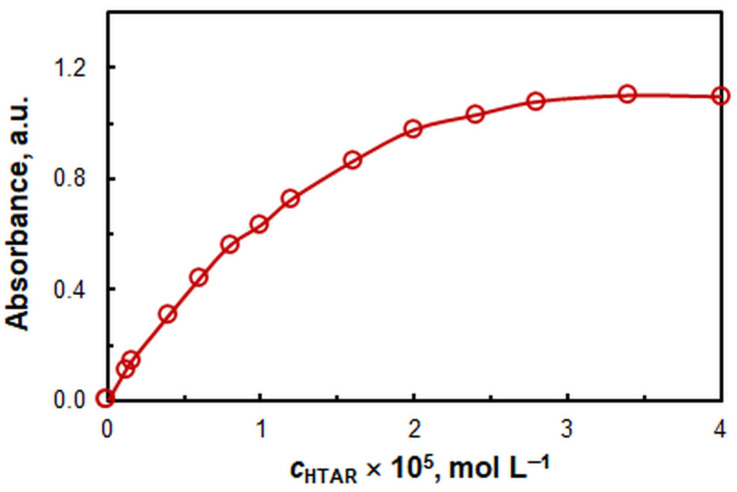
Effect of HTAR concentration. *c*_Zn(II)_ = 4 × 10^−6^ mol L^−1^, *w*_TX-114_ = 1.6%, pH 9.4, *t* = 30 min at 50 °C, *m*_SRP_ = 5 g, *λ* = 553 nm.

**Figure 3:**
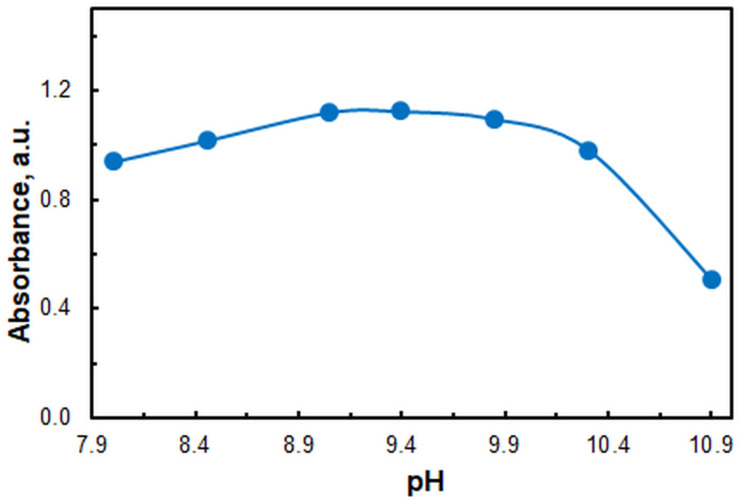
Effect of pH. *c*_Zn(II)_ = 2.4 × 10^−6^, *c*_HTAR_ = 2.8 × 10^−5^ mol L^−1^, *w*_TX-114_ = 1.2%, *V*_buffer_ = 3.0 mL, *t* = 30 min at 50 °C, *m*_SRP_ = 3 g, *λ* = 553 nm.

**Figure 4:**
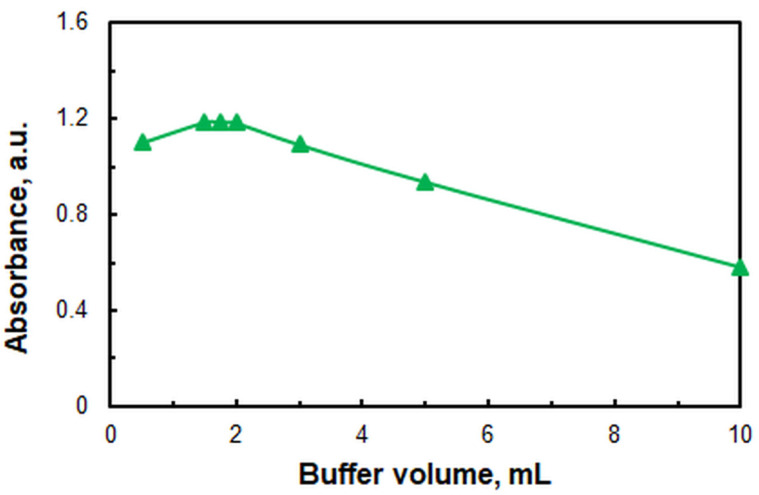
Effect of ammonium acetate buffer (pH 9.4) volume. *c*_Zn(II)_ = 2.4 × 10^−6^, *c*_HTAR_ = 2.8 × 10^−5^ mol L^−1^, *w*_TX-114_ = 1.2%, *t* = 30 min at 50 °C, *m*_SRP_ = 3 g, *λ* = 553 nm.

**Figure 5:**
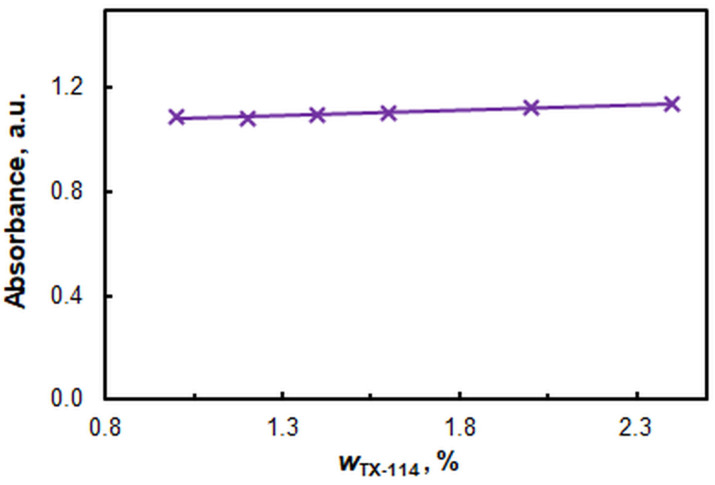
Effect of TX-114 mass fraction. *c*_Zn(II)_ = 2.4 × 10^−6^, *c*_HTAR_ = 2.8 × 10^−5^ mol L^−1^, pH = 9.4, *V*_buffer_ = 1.8 mL, *w*_TX-114_ = 1.2%, *t* = 30 min at 50 °C, *m*_SRP_ = 3 g, *λ* = 553 nm.

**Figure 6:**
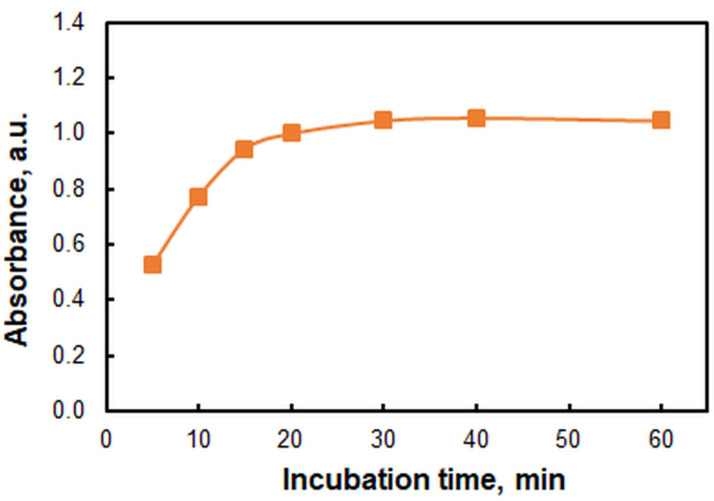
Effect of incubation time at 50 °C. *c*_Zn(II)_ = 2.4 × 10^−6^, *c*_HTAR_ = 2.8 × 10^−5^ mol L^−1^, pH = 9.4, *V*_buffer_ = 1.8 mL, *w*_TX-114_ = 1.2%, *m*_SRP_ = 3 g, *λ* = 553 nm.

In order to identify the optimal conditions for CPE–spectrophotometric analysis (i.e., the conditions for obtaining the maximum analytical signal), a single-factor optimization approach was employed. The influence of the following variables was evaluated: concentration of HTAR, pH, amount of buffer, mass fraction of TX-114, incubation time, cooling time, mass of the final surfactant-rich phase (SRP) subjected to spectrophotometry, and wavelength of the spectrophotometric measurement. The optimal conditions identified are presented in Table 1, and selected optimization steps are illustrated in Figure 2, Figure 3, Figure 4, Figure 5 and Figure 6.

Figure 2 illustrates the impact of the HTAR concentration. The optimal value is 2.8 × 10^−5^ mol L^−1^. It is not recommended to work with higher HTAR concentrations, as this can result in significant blank absorbance values.

The CPE was conducted in the presence of an ammonium acetate buffer prepared from 2 mol L^−1^ solutions of ammonia and acetic acid. As illustrated in Figure 3, the absorbance is maximal within the pH range of 9.0 to 9.8. This range is sufficiently broad to ensure reliable results. Further studies were conducted using a pH 9.4 buffer. At this pH value, the buffering capacity is at its maximum [46].

In other HTAR-based CPE systems for the determination of metal ions—for example, Cu(II) [41] and Co(II) [42]—the addition of high amounts of the same ammonium acetate buffer was found to have no effect on the absorbance. As illustrated in Figure 4, the absorbance of the extracted Zn(II) species is contingent upon the volume of buffer added (*V*_buff_). The absorbance is observed to be maximal at volumes within the range of 1.5 to 2.0 mL. Consequently, the optimal *V*_buff_ is determined to be 1.7–1.8 mL.

Figure 5 illustrates the impact of the mass fraction (*w*) of TX-114. It can be observed that the absorption remains nearly constant throughout the interval under study. It is challenging to work at *w* < 1%, as the surfactant-rich layer remains permanently turbid. The recommended *w*_TX-114_ is 1.2%.

Figure 6 illustrates the impact of incubation time at a fixed water-bath temperature of 50 °C. The time was measured from the moment the cloud-point temperature was reached, which could be easily identified visually. It is evident from Figure 6 that the incubation time (*t*) is a non-critical parameter. Further studies were conducted at *t* = 30 min.

The cooling for phase separation can be conducted at approximately −20 °C for a period of 30 to 60 min. It is not necessary to use a centrifuge. A shorter cooling time results in insufficiently viscous SRP, which interferes with phase separation by inverting the tube. If cooled for more than one hour, the upper aqueous phase may freeze.

Two approaches were evaluated for the processing of SRP:

(1) Topping up to 5.00 g with water, gently heating and shaking to obtain a homogeneous solution [40,41,42,47].
(2) Topping up to 3.00 g with a mixture of water and alcohol (ethanol or methanol).

**Figure 7:**
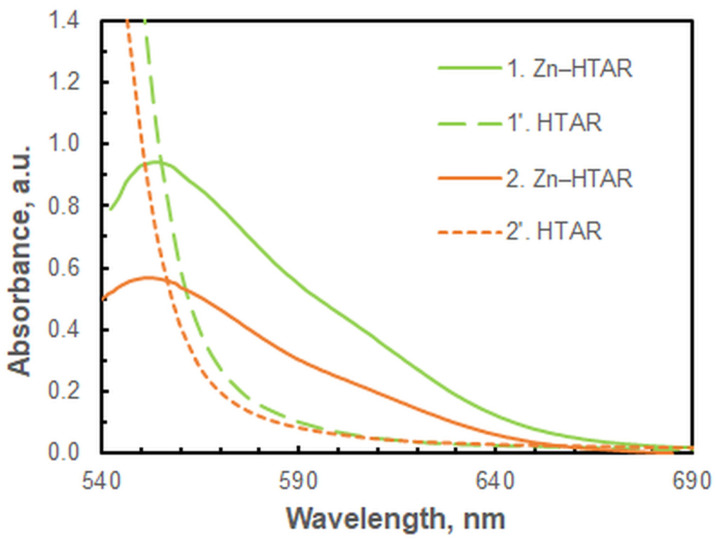
Absorption spectra of the complex species (1 and 2) and the blanks (1′ and 2′) in two different ways of SRP diluting; *c*_Zn(II)_ = 2 × 10^−6^ mol L^−1^, *c*_HTAR_ = 2.8 × 10^−5^ mol L^−1^, *w*_TX-114_ = 1.2%, pH = 9.1, *V*_buffer_ = 1.8 mL, *t* = 30 min. (1, 1′) *m* = 3.00 g, SRP diluted with ethanol (0.5 mL) and water. (2, 2′) *m* = 5.00 g; SRP diluted with water only.

The second approach is more rapid, more convenient, and provides higher sensitivity. A series of water–alcohol mixtures with varying alcohol contents was utilized to ascertain the optimal proportion. It was determined that the presence of 0.5 mL of ethanol was sufficient to produce a homogeneous liquid. Consequently, the subsequent experiments were conducted by diluting the SRP with 0.5 mL of ethanol and adding water to a total mass of 3.00 g. Figure 7 demonstrates that the location of the spectral bands is not affected by the added ethanol.

### 2.2. Stoichiometry and Suggested Structures

**Figure 8:**
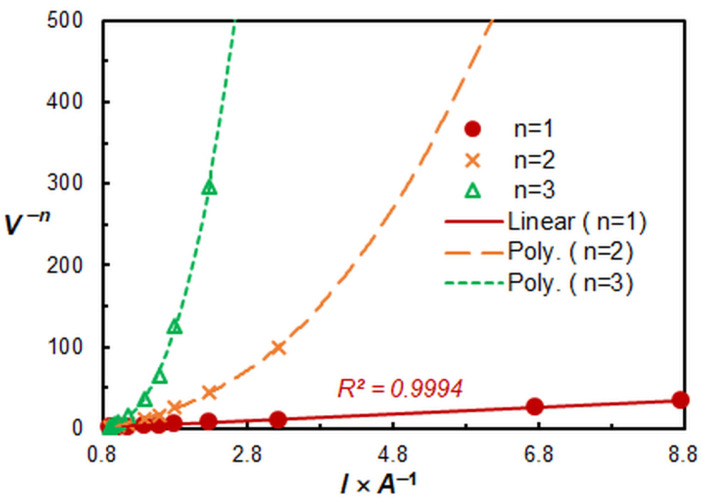
Determination of the HZH:Zn(II) molar ratio by the straight-line method of Asmus.

**Figure 9:**
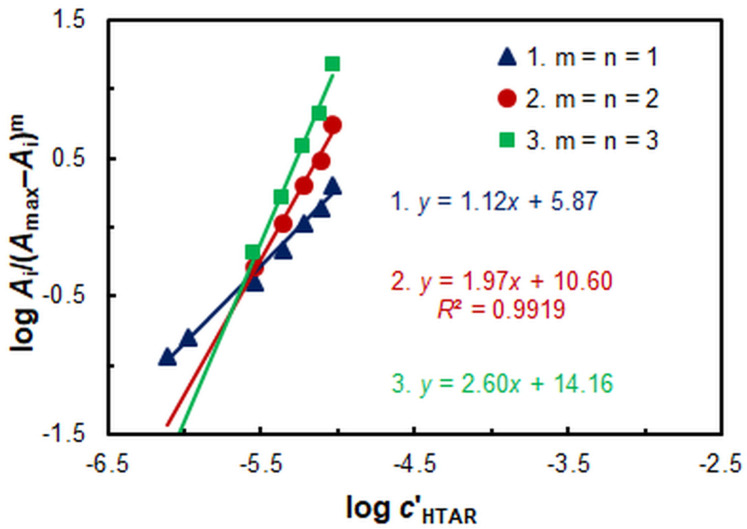
Determination of the HZH:Zn(II) molar ratio by the mobile equilibrium method.

The saturation curve presented in Figure 2 served as the basis for determining the stoichiometry of the extracted species. The results obtained by the straight-line method of Asmus [48] (Figure 8) and by the Bent and French method [49] indicate that the molar ratio of Zn(II) to HTAR is 1:1. However, these methods are unusable for species of the type M_m_R_n_, where *m* = *n* > 1. Consequently, the mobile equilibrium method [50] (Figure 9), which is capable of handling such complexes, was employed as a subsequent step. The resulting slopes of the straight lines plotted in Figure 9 are consistent with the conclusion that the complex stoichiometry is 2:2.

The chemical interactions that lead to the formation of hydrophobic extractable species are influenced by a number of factors. Among these are the ligand(s) design and the provision of an appropriate coordination number, as well as the possibilities of dimerization, which increases the hydrophobicity [51]. Zinc’s coordination numbers are usually between four and six, with three, seven, and eight being less common [52]. The tetrahedral coordination geometry is the most prevalent [53].

The HTAR, like other azoresorcinols [16,44,54], is a tridentate ligand [51]. This implies that at a 1:1 stoichiometry (Zn:HTAR), the coordination number of the complex should be 3. 

The coordination sphere could be augmented with water molecules, but this would compromise the hydrophobicity. Another potential avenue for achieving a higher coordination number is the incorporation of ammonia molecules. This assumption is supported by the observation that the optical properties of the extract are influenced by the amount of buffer added (see Figure 4).

It is conceivable that the experimentally observed 2:2 structure resulted from the dimerization of two stable 1:1 species. Given that this is an equilibrium process, the coexistence of both 2:2 and 1:1 species can be anticipated.

**Figure 10:**
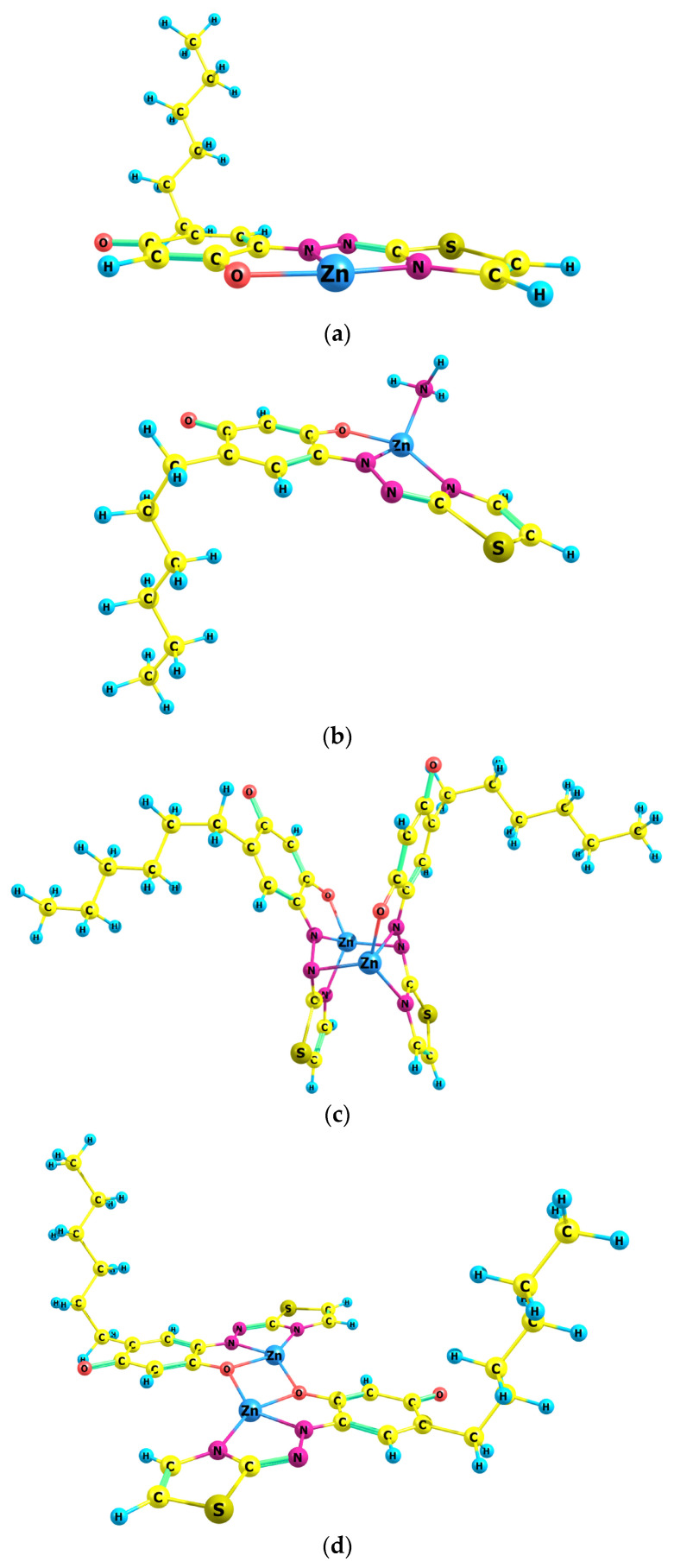
B3LYP/6-31G-optimized ground-state equilibrium geometries of the four possible structures: (**a**) Structure 1; (**b**) Structure 2; (**c**) Structure 3; (**d**) Structure 4.

A preliminary step to verify the above assumptions was to model several potential structures with a stoichiometry of 1:1 and 2:2. Their ground-state equilibrium geometries, optimized at the theoretical B3LYP/6-31G level, are presented in Figure 10. Structures 3 and 4 were obtained by pairing Structure 1 in two different ways, with consideration given to previous experience [51,54].

In Structure 1, all atoms, with the exception of the hydrophobic hexyl tail of HTAR, are arranged in a plane—a configuration also observed in the free ligand [40] and its vanadium(V) complex [55]. The polygon exhibits T-shaped symmetry with valence angles of 82.9° (N_thiazole_, Zn, N_azo_), 86.5° (N_azo_, Zn, O), and 169.5° (O, Zn, N_thiazole_). In Structure 2, which contains an additional ammonia molecule, the planarity is slightly disrupted. The polygon is a highly distorted tetrahedron with valence angles of 154.0° (O, Zn, N_thiazole_) and 137.5° (N_azo_, Zn, N_ammonia_). The planarity of the two ligands in the dimeric Structure 3 is most significantly disrupted. The two zinc atoms are also tetra-coordinated. In this structure, a six-membered ring involving the Zn atoms and the nitrogen atoms of the azo groups is observed. Structure 4 appears to be more stable than Structure 3. In it, the planarity of the ligands is largely preserved, and the oxygen atoms in the ortho position relative to the azo groups act as bridge atoms between the two coordination centers.

### 2.3. Comparison of Theoretical and Experimental Spectra

**Figure 11:**
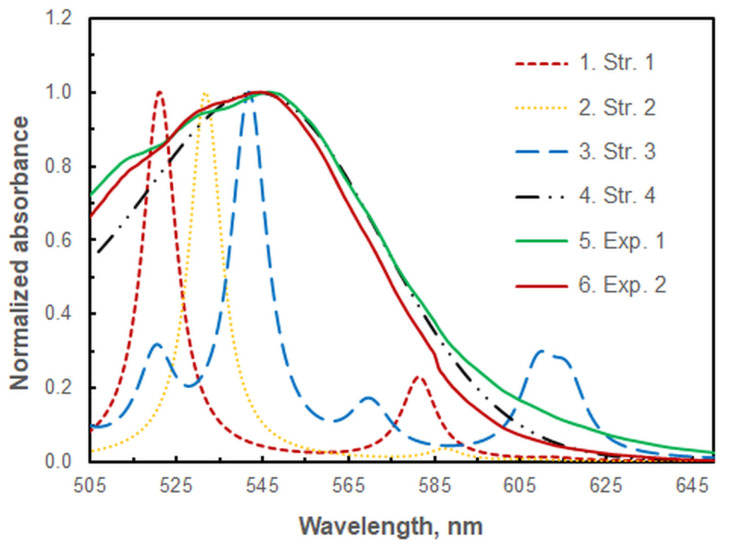
Comparison of the four theoretical spectra (Structures 1–4) at the B3LYP/6-31G level of theory and a scaling factor of 1.136 with two experimental spectra (Exps. 1 and 2) recorded at different buffer volumes: 1.8 mL (Exp. 1) and 10 mL (Exp. 2). *c*_Zn(II)_ = *c*_HTAR_ = 6 × 10^−6^ mol L^−1^, *w*_TX-114_ = 1.2%, pH = 9.4, *t* = 30 min at 50 °C, *m*_SRP_ = 3 g.

The structures depicted in Figure 10 were used to compute vertical excitation energies with the time-dependent Hamiltonian, with the objective of simulating their theoretical absorption spectra. Figure 11 compares these spectra with two experimental spectra (Exp. 1 and Exp. 2) obtained using different volumes of the buffer with the optimum pH of 9.4. It can be concluded that Exp. 1, obtained at the optimal buffer volume, is closer to the theoretical spectrum of Structure 4. However, both experimental spectra demonstrate the presence of the other three structures (Structures 1–3). For instance, the kink at approximately 585 nm in Exp. 2 suggests that Structure 1 is formed even at high ammonia concentrations.

### 2.4. Energy Analysis of the Dimeric Structures

The shoulder observed at approximately 610 nm in the experimental spectra at the optimum HTAR concentration (Figure 7), along with the spectra presented in Figure 1 and Figure 11, lead to the conclusion that Structure 3 exists in the extract, along with the dominant Structure 4. A comparison of the energies of the two binuclear complexes (Structure 3 and Structure 4) is therefore warranted. The calculated energy quantities at the selected theoretical level indicate that Structure 4 is approximately 175 kJ mol^−1^ more stable than Structure 3. Additionally, the transformation from Structure 3 to Structure 4 is accompanied by a negative free energy change, ΔG = −179 kJ mol^−1^, and a heat effect, ΔG = −172 kJ mol^−1^.

### 2.5. Extraction Constant and Fraction Extracted

Given that under optimal CPE conditions predominantly 2:2 species are extracted, the mobile equilibrium method (Figure 9, line 2) can be employed to calculate the conditional extraction constant (*K*_ex_). The determined logarithmic value of this constant is log *K*_ex_ = 15.9 ± 0.5. In the calculations, the recommendation of the authors of the method [50] was considered, which advised the use of the abscissa intercept in lieu of the ordinate intercept.

The fraction extracted (%*E*) was calculated to be (98.3 ± 1.4) %. This value is indicative of the quantitative extraction of Zn(II).

### 2.6. Calibration Curve and Analytical Characteristics

**Figure 12:**
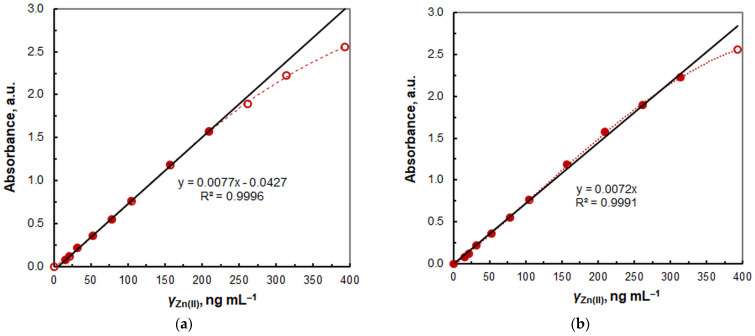
Calibration graph for determination of Zn(II). The experimental conditions are given in Table 1. In panel (**a**), the intercept is statistically different from zero, whereas in panel (**b**), it is set to zero.

The relationship between absorbance and zinc concentration under optimal CPE conditions was linear within the range of 15.7 to 209 ng mL^−1^ Zn(II) (Figure 12a), with a remarkable squared correlation coefficient (*R*^2^ = 0.9996). The linear regression equation was found to be *A* = 7.748*γ* − 0.0427, where *γ* represents the Zn(II) mass concentration in μg mL^−1^. The standard deviations of the slope and intercept were 0.065 and 0.0068, respectively. The intercept is relatively small but statistically distinguishable from zero. This can be attributed to the fact that at a low Zn(II) concentration, the presence of the minor 1:1 species is more noticeable.

If the intercept is set to zero (Figure 12b), the relationship becomes linear up to 314 ng mL^−1^ Zn(II), with an *R*^2^ value of 0.9991. The resulting line is then described by the equation *A* = 7.243*γ*. The calculated molar absorptivity coefficient (*ε*) is 4.47 × 10^5^ L mol^−1^ cm^−1^, and the Sandell sensitivity is 0.138 ng cm^−2^. The limit of detection (LOD) and quantitation (LOQ) were calculated as 3 and 10 times the standard deviation of the blank divided by the slope, resulting in values of 4.8 ng mL^−1^ and 16 ng mL^−1^, respectively. The obtained LOQ value is in close proximity to the lowest concentration (15.7 ng mL^−1^) at which Beer’s law is observed with an *R*^2^ value of 0.9996 (Figure 12a).

The density of the final SRP in the presence of 0.5 mL of ethanol, determined at room temperature with a pycnometer, was found to be close to unity (*ρ* = 0.99). Consequently, the preconcentration factor, calculated as the ratio of the highest sample volume (50 mL) to the lowest final volume (3.00 g = 3.03 mL), was determined to be 16.5.

A comparable value (PF = 16.4) was attained by dividing the slopes of the calibration lines obtained after CPE and without CPE (in the absence of TX-114 and without heating). The lack of a significant discrepancy between these values indicates that the chemistry of the complexation process is unlikely to be altered upon heating.

### 2.7. Impact of Foreign Ions

The influence of foreign ions is presented in Table 2. The presence of large amounts of common ions, such as Na^+^, K^+^, Cl^−^, SO_4_^2−^, NO_3_^−^, and F^−^, is tolerable, which does not place restrictions on how the samples are dissolved. Ions such as Mo(VI), W(VI) and Re(VII) also do not interfere in large amounts. The most significant interferences are caused by Cd(II), Co(II), Cu(II), Ni(II) and Pb(II), which form well-extractable complexes under the experimental conditions.

### 2.8. Analytical Application

The method was applied to determine the zinc content of Zinkorot^®^ tablets, which are claimed to contain 25 mg of zinc per tablet. The result was statistically identical to this value: 24.7 ± 0.8 (mean ± SD, four replicate analyses). To evaluate the inter-day reproducibility, eight replicate analyses of the solution were performed on the next two days (four analyses per day). The pooled results for the three consecutive days demonstrated that the reproducibility was satisfactory, with a mean value of 24.8 and a relative standard deviation (RSD) of 3.7%.

The range of analyses was subsequently expanded by processing a dermal ointment (containing 10% ZnO) and two industrial samples, namely zinc concentrate and granulated zinc powder. The results are presented in Table 3. The RSD was in the range of 2.5–3.1%.

### 2.9. Comparison with Existing Methods

Table 4 presents a summary of the data for the CPE–spectrophotometric methods for the determination of Zn(II). The CL-CPE method can be labelled as simple, convenient, inexpensive, sensitive, and ecologically friendly. The reagent is commercially available and does not require synthesis, in contrast to the procedures described in Refs. [23,24,35,56]. The incubation temperature (50 °C) is lower than that applied when using TX-100 (80–95 °C). The fraction extracted is significant, as the complex species are hydrophobic. Therefore, it is not necessary to add electrolytes to increase the fraction extracted. The method is not encumbered by absorption–desorption processes, as in Ref. [34], or by the mathematical treatment of absorbances at different wavelengths, as in Ref. [33]. 

One limitation of the proposed method is the lack of selectivity for certain side ions, which should be considered when selecting samples for analysis. Additionally, the absorbance of the blank at *λ*_max_ is relatively high. However, this drawback can be easily overcome by measuring at a longer wavelength, if necessary.

**Table 4:** Comparison with published CPE–spectrophotometric methods for Zn(II) determination.

Reagent(s)	Technique	Surfactant	Acidity	λ, nm	Linear Range, ng mL^−1^	Sample	Ref.
PAN	CPE–UV/Vis	PONPE-7.5	pH 10.0	555	2–60	Tap water	[18]
PAN	CPE–UV/Vis, PLSR	TX-114	pH 9.2	550	2–150	Water and urine samples	[33]
TiO_2_ NPs + dithizone	CPE–SPE–UV/Vis	TX-100	pH 7.0	530	0.5–90	Tap water, powder milk, and zinc sulfate tablet	[34]
PANN	CPE–UV/Vis	TX-100	1 mol L^−1^ HCl	414	100–3000	Vegetables, meet, and water samples	[35]
Na_2_EDTA + BG	CPE–UV/Vis	TX-100	pH 10.0	630	100–10,000	Food samples	[38]
HTAR	CL-CPE–UV/Vis	TX-114	pH 9.4	554	15.7–209	Pharmaceutical and industrial samples	This work

Abbreviations: BG, Brilliant Green; Na_2_EDTA, ethylenediaminetetraacetic acid disodium salt dihydrate; NPs, nanoparticles; PANN, 3-[(2-pyridyl azo)]-1-nitroso-2-naphthol; PAN, 1-(2-pyridylazo)-2-naphthol; PLSR, Partial least-squares regression; PONPE-7.5, polyoxyethylene nonyl phenyl ether; SPE, solid phase extraction; TX-100, Triton X-100; TX-114, Triton X-114.

## 3. Materials and Methods

### 3.1. Instrumentation and Chemicals

A Spectronic Camspec M550 and Ultrospec 3300 pro scanning UV-Vis spectrophotometers (Garforth, UK) equipped with 1 cm cuvettes were used for spectrophotometric measurements. The pH was determined using a WTW InoLab 7110 pH meter (Weilheim, Germany). An Ohaus Pioneer PA214C top-loading analytical balance (Parsippany, NJ, USA) was used for mass quantification. Samples were heated in a GFL 1023 water bath (Berlin, Germany).

The chemicals were of the Merck brand. The stock Zn(II) solution (1 g L^−1^) was prepared by dissolving high-purity zinc with an appropriate amount of HCl (1:1) [28,38,57]. Further dilution was employed to prepare working solutions with a concentration of 2 × 10^−4^ mol L^−1^. The HTAR was dissolved in the presence of potassium hydroxide, resulting in an aqueous solution with a concentration of 2 × 10^−3^ mol L^−1^ [39]. Laboratory-grade TX-114, diluted with water at a mass fraction of 10%, was utilized. A series of buffer solutions was prepared by mixing the appropriate volumes of aqueous solutions of 2 mol L^−1^ acetic acid and 2 mol L^−1^ ammonia. The experiments were conducted using distilled water.

### 3.2. Samples and Sample Preparation

Zinkorot^®^ (Woerwag Pharma, Böblingen, Germany), a zinc supplement indicated for the treatment of zinc deficiency conditions, was procured from a local pharmacy. According to the product label, each tablet contains 25 mg of zinc. The tablets were prepared for analysis by the procedure outlined in [42], which involved heating in a mixture of concentrated nitric acid (10 mL) and concentrated sulfuric acid (1 mL) on a sand bath. 

A Zn-containing dermal ointment was prepared by mixing 10 g zinc(II) oxide with 90 g petrolatum. The mixture was then heated under agitation in a water bath at 85 °C. The resulting ointment was subjected to dissolution according to a known procedure, which entailed treatment with 0.5 mol L^−1^ nitric acid to dissolve the oxide, followed by liquid–liquid extraction with diethyl ether to separate the organic component [58]. 

The industrial samples (granulated dust and zinc concentrate) were provided by KCM SA (Plovdiv, Bulgaria), a metallurgical smelter engaged in the production of zinc and other metals. The samples were dissolved in concentrated nitric acid, and the resulting solutions were transferred to 1000 mL flasks, with water added until the mark.

### 3.3. CPE–Spectrophotometric Optimization

Solutions of TX-114, Zn(II), ammonium acetate buffer, and HTAR were transferred in a sequential manner into conical centrifuge tubes of known mass. The resulting mixtures were diluted to 50 mL with water and heated in a water bath at 50 °C for a fixed period of time. Subsequently, the tubes were cooled in a refrigerator to ensure completion of the precipitation process and to facilitate removal of the supernatant by inverting the tubes. Then, a mixture of alcohol and water was added to the remaining SRPs, resulting in a total mass (SRP + alcohol + H_2_O) of 3.00 g in each case. Finally, the mixtures were homogenized by gentle shaking. 

An alternative dilution procedure for the SRPs was as described in [40,41,42]. In this procedure, only water was added to a final mass of 5.00 g (SRP + H_2_O). The samples were then homogenized by heating (for 1–2 min at 40–45 °C) and shaking for several minutes.

Irrespective of the manner in which the SRP was processed, a portion of the final clear solution was transferred to a cuvette, and the absorbance was measured against a simultaneously prepared blank. 

### 3.4. Determination of the Fraction Extracted

The fraction extracted was determined via the formula %*E* = 100 × *A*_1_/*A*_3_, where *A*_1_ and *A*_3_ are the corresponding absorbances for a single and a triple extraction (*n* = 5) at the optimum CPE conditions in equal final masses of the SRP (10.00 g).

### 3.5. Recommended Procedure for the Determination of Zinc(II)

A 50 mL conical tube was weighed using a top-loading analytical balance, and 6 mL of a 10% Triton X-114 solution was placed therein. Subsequently, an aliquot of the analyzed solution (15.7–209 ng mL^−1^ of Zn), 1.8 mL of a buffering solution with a pH of 9.4, and 0.7 mL of a 2 × 10^−3^ molar solution of HTAR were added. The tube was filled to a total volume of 50 mL with water, and the mixture was heated for 30 min in a water bath at 50 °C. After cooling in a refrigerator (at approximately −20 °C) for 40 min, at which point the precipitation process was complete and the SRP was virtually solid, the supernatant was removed by inverting the tube. Subsequently, 0.5 mL of ethanol and water were added to the SRP, resulting in a total mass of 3.00 g (the empty tube mass data were used for this operation). Following brief shaking to homogenize the solution, it was transferred to a cuvette. The absorbance was then measured at 553 nm against a similarly prepared blank. 

### 3.6. Theoretical Section

The ground-state equilibrium geometries of the four suggested complexes were optimized at the B3LYP/6-31G level of theory in the gas phase, with no symmetry or structural restrictions. The spin multiplicity and charge were set to 1 and 0, respectively. Subsequent frequency calculations were performed to prove that the found geometries are located in minima on the hypercoordinate potential energy surface (PES). Additionally, the vertical excitation energies of the systems were calculated in order to simulate their UV spectra and facilitate a comparison with the experimental data. The calculations were conducted using the GAUSSIAN 03 software [59]. The ChemCraft 1.8 program [60] was employed for the visualization of the structures.

## 4. Conclusions

A CL-CPE–chromogenic system for Zn(II) ions was investigated. The system is based on the hydrophobic azo dye HTAR and the non-ionic surfactant TX-114. The complex formation occurs in a weakly alkaline medium (ammonium acetate buffer), and the four complexes that were detected have a stoichiometry of 1:1 (Zn:HTAR), 1:1:1 (Zn:HTAR:NH_3_), and 2:2 (Zn:HTAR; two different species, which differ by the mutual arrangement of the ligands and the type of bridging atoms). In the optimal extraction conditions, the dominant species is a binuclear complex with oxygen-bridging atoms. This complex serves as the basis for a simple, convenient, cost-effective, sensitive, and environmentally friendly CL-CPE method for the spectrophotometric determination of Zn(II).

## Figures and Tables

**Table 1:** Optimization of the CPE system.

Optimized Parameter	Investigated Range	Optimal Value
Concentration of HTAR, mol L^−1^	(0.12–4) × 10^−5^	2.8 × 10^−5^
pH	8.0–10.9	9.4
Volume of the buffer, mL	0.5–10	1.8
Mass fraction of TX-114, %	0.6–2.4	1.2
Incubation time at 50 °C, min	5–60	30
Cooling time at −20 °C, min	20–70	40
Wavelength, nm	Visible range	553

**Table 2:** Impact of foreign ions on the determination of 3.9 μg Zn(II).

Foreign Ion (FI)	Formula of the Salt	Mass of the FI, μg	FI:Zn(II) Mass Ratio	Mass of Zn Found, μg	*E*, %
Al(III)	Al(NO_3_)_3_·9H_2_O	39	10	4.1	106
Ba(II)	Ba(NO_3_)_2_	3900	1000 *	4.0	101
Ca(II)	Ca(NO_3_)_2_	1950	500	4.1	106
Cd(II)	CdCl_2_	2.0	0.5	**7.2**	127
Co(II)	Co(SO_4_)_2_·7H_2_O	2.0	0.5	5.9	151
Cr(III)	Cr_2_(SO_4_)_3_	3.9	1	3.9	99.2
Cr(VI)	K_2_CrO_4_	19.5	5	4.0	103
Cu(II)	CuSO_4_·5H_2_O	2.0	0.5	4.8	123
F^−^	NaF	19,500	5000	3.7	95.8
Fe(III)	Fe_2_(SO_4_)_3_	19.5	5	3.8	96.4
HPO_4_^2−^	Na_2_HPO_4_·12H_2_O	780	200	3.8	98.3
Hg(II)	Hg(NO_3_)_2_	78	20	3.9	100
Mg(II)	MgSO_4_·7H_2_O	1950	500	3.9	100
Mn(II)	MnSO_4_·H_2_O	7.8	2	3.9	99.0
Mo(VI)	(NH_4_)_6_Mo_7_O_24_·4H_2_O	3900	1000 *	4.2	106
Na(I)	NaCl	39,000	10,000 *	4.0	102
Ni(II)	NiSO_4_·7H_2_O	2.0	0.5	4.1	105
NO_3_*^−^*	NH_4_NO_3_	39,000	10,000 *	3.9	100
Re(VII)	NH_4_ReO_4_	3900	1000 *	4.1	106
Pb(II)	Pb(NO_3_)_2_	3.9	1	4.8	123
SO_4_^2−^	K_2_SO_4_	39,000	10,000 *	3.9	100
V(V)	NH_4_VO_3_	7.8	2	4.0	104
W(VI)	Na_2_WO_4_·2H_2_O	3900	1000 *	3.8	98.0

* Higher FI:Zn ratios have not been studied.

**Table 3:** Determination of zinc in pharmaceutical and industrial samples.

#.	Sample	Zn Mass Fraction (*w*_Zn_), %	Other Ingredients	*w*_Zn_ Found ^b,c^, %
1	Dermal ointment	8.03	90% petrolatum	8.0 ± 0.2
2	Granulated zinc dust	19.5 ^a^	26.9% Fe, 7.9% CaO, 5.45% SiO_2_, 2.57% Pb, 2.18% Cl, 1.78% MgO, 1.02% S, 0.53% Al, 0.24% Cu, 0.22% F, 0.07% Cd, 0.013% Sb, 0.009% As, 0.002% Co, 29 μg g^−1^ Tl, 22 μg g^−1^ Se, 14 μg g^−1^ Te, 10 μg g^−1^ Ge ^a^	19.8 ± 0.6
3	Zinc concentrate	49.7 ^a^	31.75% S, 1.75% SiO_2_, 1.1% Pb, 0.32% Al_2_O_3_, 0.24% Cd, 0.17% Cu, 0.08% CaO, 0.071% Sb, 0.07% MgO, 0.016%As, 0.01% Cl, 0.005% F, 0.002% Co, 0.001% Ni, 11 μg g^−1^ Tl, 10 μg g^−1^ Ge, 5 μg g^−1^ Se, 5 μg g^−1^ Te, 3 μg g^−1^ Hg ^a^	49.1 ± 1.4

^a^ Determined in another laboratory. ^b^ Mean ± standard deviation. ^c^ Four replicate determinations.

## Data Availability

Data are contained within the article.
